# Multigene phylogenetic analyses and morpho-anatomical characterization revealed two new species of Xylariomycetidae from Yunnan, China

**DOI:** 10.3897/mycokeys.127.179422

**Published:** 2026-01-29

**Authors:** Ming-Hui He, Xing-Yu Luo, Kamran Habib, Ping-Zhu Lu, Yi-Lan Jin, Urooj Ashraf, Qi-Rui Li

**Affiliations:** 1 State Key Laboratory of Discovery and Utilization of Functional Components in Traditional Chinese Medicine & School of Pharma-ceutical Sciences, Guizhou Medical University, Guian New District, Guizhou 550004, China Anshun University Agricultural College Anshun China; 2 The High Efficacy Application of Natural Medicinal Resources Engineering Centre of Guizhou Province (The Key Laboratory of Optimal Utilization of Natural Medicine Resources), School of Pharmaceutical Sciences, Guizhou Medical University, Guiyang, Guizhou, 550004, China Institute of Botany, University of the Punjab Lahore Pakistan; 3 Chongqing Three Gorges Medical College, Wanzhou Chongqing, 404100, China State Key Laboratory of Discovery and Utilization of Functional Components in Traditional Chinese Medicine & School of Pharma-ceutical Sciences, Guizhou Medical University Guizhou China; 4 Anshun University Agricultural College, Anshun, Guizhou province, 561000, China School of Pharmaceutical Sciences, Guizhou Medical University Guizhou China; 5 Fungal Biology and Systematics Laboratory, Institute of Botany, University of the Punjab, Quaid-e-Azam Campus-54590, Lahore, Pakistan Chongqing Three Gorges Medical College Wanzhou Chongqing China

**Keywords:** Two novel taxa, bambusicolous fungi, Karst ecosystem, Southwestern China

## Abstract

During a survey of ascomycetes in Yunnan Province, China, two new wood-inhabiting fungi were collected and identified. These novel species are described based on morphological characteristics and phylogenetic analyses of the ITS, LSU, *rpb2*, and *tef1* loci. The newly identified species are designated as *Amphisphaeria zhaotongensis* and *Pallidoperidium yunnanense*, belonging to the families Amphisphaeriaceae and Pallidoperidiaceae, respectively. These species are confirmed as distinct from their close relatives through phylogenetic analyses and morpho-anatomical comparison. The study provides detailed morphological descriptions, illustrative representations, and a phylogenetic tree, all of which contribute to the taxonomic positioning of these novel species.

## Introduction

Yunnan Province, located in southwestern China, is a globally recognized biodiversity hotspot. Its complex topography and range of habitats, including extensive karst systems and montane forests, support remarkably high levels of species richness and endemism (Xu et al. 2017). Mycological research in Yunnan has accelerated markedly in the last decade, with reports focusing on areas such as fungal secondary metabolites, ethno-mycology, and the taxonomy of microfungi and macrofungi using morpho-molecular approaches (Hongsanan et al. 2023). Systematic mycological surveys are essential, not only to uncover misidentified or poorly known species but also to reveal undescribed diversity. Such deeper exploration of fungal richness can also provide valuable insights into ecological interconnections and overall ecosystem health (Wijayawardene et al. 2021; Dissanayake et al. 2024; Habib et al. 2025; Liu et al. 2025).

In this publication, we describe two new species belonging to the subclass Xylariomycetidae, representing the families Amphisphaeriaceae and Pallidoperidiaceae. Amphisphaeriaceae was introduced by Winter (1887, as “Amphisphaerieae”) to accommodate the type genus *Amphisphaeria* Ces. & De Not. and related taxa. Members of Amphisphaeriaceae are predominantly saprobes on decaying wood in terrestrial, marine, and freshwater habitats, although some taxa are hemibiotrophic or necrotrophic (Wang et al. 2004; Senanayake et al. 2015, [Bibr B33][Bibr B13]). According to the latest Outline of Fungi (Hyde et al. 2024), Amphisphaeriaceae presently contains the two genera *Amphisphaeria* and *Labridella* Brenckle. *Amphisphaeria* is the type genus of the family, with *A. umbrina* (Fr.) De Not. designated as the type species (Cesati and De Notaris 1863). Species of *Amphisphaeria* are known to produce both coelomycetous and hyphomycetous asexual morphs and have saprobic and endophytic lifestyles, commonly on woody substrates and some monocot hosts including grasses. (Samarakoon et al. 2020, 2022; Wang et al. 2023; Dissanayake et al. 2024). The teleomorph of the genus is characterized by solitary or aggregated ascomata under a poorly-developed clypeus or lack of clypeus; unitunicate asci with an apical apparatus that is amyloid or inamyloid, and light brown to dark brown, ellipsoid to fusiform, 1–3-septate ascospores (Cesati and De Notaris 1863; Pathirana et al. 2025; Samarakoon et al. 2020; Samarakoon et al. 2023). According to Species Fungorum (accessed 28 September 2025), the genus currently comprises 169 species. Pallidoperidiaceae was established by Sugita et al. (2024) to accommodate a distinct, monophyletic lineage of Anthostomella-like fungi associated with bamboo. Sugita et al. (2024) included five genera (*Amphigermslita* R. Sugita & Kaz. Tanaka, *Crassipseudostroma* R. Sugita & Kaz. Tanaka, *Minuticlypeus* R. Sugita & Kaz. Tanaka, *Pallidoperidium* R. Sugita & Kaz. Tanaka, and *Nigropunctata* M.C. Samar. & K.D. Hyde) in the family. Recently, Lu et al. (2025) included *Melanographium*, previously considered incertae sedis, within Pallidoperidiaceae based on multigene phylogenetic analyses and concordance in teleomorph characters. Pallidoperidiaceae is typified by the genus *Pallidoperidium*, of which *P. exasperatum* R. Sugita & Kaz. Tanaka is the type species. The genus is characterized by perithecial ascomata, immersed, solitary, with a conical to cylindrical, periphysate ostiolar neck, a thick, inconspicuous ascomatal wall composed of hyaline to pale brown cells, surrounded by pseudostromatic tissue, numerous, septate, unbranched paraphyses, unitunicate, cylindrical, 8-spored asci with a thin discoid, amyloid apical apparatus, and ellipsoid to fusiform, unicellular, brown, rough ascospores surrounded by a mucilaginous sheath and with a germ slit (Sugita et al. 2024). *Pallidoperidium* comprises four species, with *P. chinense* K. Habib et al. and *P. smilacis* (Fabre) K. Habib & Q.R. Li reported from China (Habib et al. 2025), and *P. exasperatum* R. Sugita & Kaz. Tanaka and *P. paraexasperatum* R. Sugita & Kaz. Tanaka from Japan (Sugita et al. 2024).

In a study focused on the diversity of Xylariomycetidae in Guizhou, China, we identified two species belonging to the genera *Amphisphaeria* and *Pallidoperidium*. Morphological comparisons, together with phylogenetic analyses based on ITS, LSU, *rpb2*, and *tef1* loci, showed that these taxa did not correspond to any previously described species within their respective genera. Based on this, we propose two new species, for which we provide brief diagnoses, detailed descriptions, illustrations, and phylogenetic placements.

## Materials and methods

### Sample collection and morphological study

The specimens were collected during surveys conducted in Yunnan province between 2023 and 2024, with all relevant habitat information recorded in a detailed manner. The photos of the collected materials were taken using a Canon G15 camera (Canon Corporation, Tokyo, Japan). Materials were placed in paper bags and taken to the lab for morphological characterization and isolation. To remove excess humidity, they were dried in the shade at room temperature. The dried specimens were carefully labeled and stored until further processing. All specimens were deposited at the Herbarium of Guizhou Medical University (GMB) and the Herbarium of Cryptogams, Kunming Institute of Botany, and the Chinese Academy of Sciences (KUN), while living cultures were deposited at the Guizhou Medical University Culture Collection (GMBC).

### Morphological characterization and isolation

Macroscopic features of the specimens were examined using an Olympus SZ61 stereomicroscope and photographed using a Canon 700D digital camera. Microscopic morphological features (ascomata, peridium, paraphyses, asci, ascospore), were observed using DIC and photographed using a Canon 700D digital camera attached. Melzer’s iodine reagent was used to test the apical apparatus structures for amyloid reaction. Asci and ascospores of the samples were measured using Tarosoft Image Framework (v. 0.9.0.7). Images were processed using Adobe Photoshop CS6 (Adobe Systems, USA). Pure cultures were obtained by single-ascospore isolation (Long et al. 2019) and maintained at 25 °C for 1–5 weeks on PDA (potato dextrose agar) and oatmeal-agar (OA) medium by adding Veterinary sterptpmycin, on 100,000 units per liter.

### DNA extraction, PCR amplification and sequencing

Mycelium was scraped from pure culture plates using a sterilized scalpel and used for DNA extraction using the BIOMIGA fungus genomic DNA extraction kit following the manufacturer’s instructions. For some specimens where the ascospores did not germinate, we directly extracted DNA from the contents of the perithecium. The DNA samples were kept at –20 °C. Sequences of the internal transcribed spacer (ITS), the large subunit (LSU), *rpb2* and translation elongation factor (*tef1α*) were amplified by PCR with primers ITS1/ITS4 (White et al. 1990; Gardes and Bruns 1993), LR0R/LR5 (Vilgalys and Hester 1990), Bt2a/Bt2b (Glass and Donaldson 1995), and EF1-983F/EF1-2218R (Rehner and Buckley 2005), respectively. The components of a 25 μL volume PCR mixture were: 9.5 μL of double distilled water, 12.5 μL of PCR Master Mix, 1 μL of each primer, and 1 μL of template DNA. Qualified PCR products were checked through 1.5% agarose gel electrophoresis stained with Golden View, and were sent to Sangon Co., China, for sequencing.

### Sequence alignments and phylogenetic analyses

All the obtained sequences were deposited in GenBank. Generated sequences were compared with each other and deposited sequences available through GenBank using the BLASTn. A molecular phylogeny was inferred from a combined dataset of ITS, LSU, *rpb2* and *tef1* sequences. The reference sequences retrieved from open databases originated from recently published literature (Habib et al. 2025; Dissanayake et al. 2024; Du et al. 2025; Li et al. 2024 and Sugita et al. 2024) and the BLASTn results of close matches (Table 1).

**Table 1.** Taxa and corresponding GenBank accession numbers of sequences used in the phylogenetic analyses of Figs 1, 2.

Species	Voucher	GenBank Accession Numbers				References
		ITS	LSU	*rpb2*	*tef1*	
*Amphigermslita deformis*	HHUF 30660^T^	NR190918	NG243031	LC760592	LC760605	Sugita et al. (2024)
*Amphigermslita deformis*	HHUF 30661	LC760554	LC760573	LC760593	LC760606	Sugita et al. (2024)
*Amphigermslita fusiformis*	HHUF 30663^T^	NR190919	NG243032	LC760594	LC760607	Sugita et al. (2024)
*Amphigermslita fusiformis*	HHUF 30664	–	LC760575	LC760595	LC760608	Sugita et al. (2024)
*Amphigermslita pseudofusiformis*	HHUF 30662^T^	NR190920	NG243033	LC760596	LC760609	Sugita et al. (2024)
*Amphigermslita subyunnanensis*	GMB1153	PP133235	PQ860488	–	–	Habib et al. (2025)
*Amphigermslita yunnanensis*	HKAS 122747	OQ158966	OQ170888	–	–	Habib et al. (2025)
*Amphisphaeria acericola*	MFLUCC 14-0842	MF614128	MF614131	–	–	Senanayake et al. (2019)
*Amphisphaeria acericola*	MFLU 16-2479	MT756624	MK640424	–	–	Senanayake et al. (2019)
*Amphisphaeria ailaoshanensis*	KUNCC 23-15520^T^	PP584673	PP584770	–	–	Dissanayake et al. (2024)
*Amphisphaeria ailaoshanensis*	KUNCC 23-15521	PP584674	PP584771	–	–	Dissanayake et al. (2024)
*Amphisphaeria camelliae*	HKAS 107021^T^	MT756621	MT756615	MT789850	MW759530	Samarakoon et al. (2020)
*Amphisphaeria camelliae*	MFLU 20-0181	MT756622	MT756616	MT789851	–	Samarakoon et al. (2020)
*Amphisphaeria chiangmaiensis*	CMUB 40017	OR507139	OR507152	OR504416	OR504423	Samarakoon et al. (2023)
*Amphisphaeria curvatoconidia*	MFLU 18-0789^T^	MT756623	MT756617	–	MW759529	Samarakoon et al. (2020)
*Amphisphaeria curvatoconidia*	HKAS 102288	MT756624	MT756618	MT789853	–	Samarakoon et al. (2020)
*Amphisphaeria davidihuangtciae*	UESTCC 23-0548	PQ773396	PQ773416	PV075242	–	Du et al. (2025)
*Amphisphaeria davidihuangtciae*	UESTCC 25-0040^T^	PQ773395	PQ773415	PV075241	–	Du et al. (2025)
*Amphisphaeria flava*	MFLUCC 18-0361^T^	MH971224	NG068587	–	MW759528	Samarakoon et al. (2019)
*Amphisphaeria fuckelii*	CBS 140409^T^	NR154123	–	MK523280	MK523308	Jaklitsch et al. (2016)
*Amphisphaeria guttulata*	MFLU 22-0078^T^	OQ101582	OQ101583	–	–	–
*Amphisphaeria hydei*	CMUB 40016	OR507141	OR507154	OR504417	OR504424	Samarakoon (2023)
*Amphisphaeria hydei*	MFLU 23-0412^T^	OR507142	OR507155	OR504418	–	Samarakoon (2023)
*Amphisphaeria_hydeimucosa*	CGMCC 3.27285	PQ189779	PQ184734	PQ379998.	-	Du et al. (2025)
*Amphisphaeria_hydeimucosa*	UESTCC:23.0462	PQ191046	PQ184721	PQ379951	-	Du et al. (2025)
*Amphisphaeria karsti*	GZAAS 20-0148^T^	OR224992	OR209623	–	–	Samarakoon et al. (2020)
*Amphisphaeria kunmingensis*	HKAS 130268	PP584675	PP584772	–	–	Dissanayake et al. (2024)
*Amphisphaeria magna*	HKAS 130270^T^	NR198753	PP584774	–	–	Dissanayake et al. (2024)
*Amphisphaeria magna*	HKAS 130271	PP584678	PP584775	–	–	Dissanayake et al. (2024)
*Amphisphaeria mangrovei*	PUFD37^T^	MG844283	MG844275	–	–	Phookamsak et al. (2019)
*Amphisphaeria oleae*	HKAS 128843	OR253156	OR253313	OR253756	–	Li et al. (2024)
*Amphisphaeria parvispora*	MFLU 18-0767	NR175677	NG081501	MW658631	MW759532	Samarakoon et al. (2022)
*Amphisphaeria qujingensis*	KUMCC 19-0186	MN707568	MN707566	–	–	Dissanayake et al. (2020)
*Amphisphaeria qujingensis*	KUMCC 19-0187^T^	NR169986	MN556316	MN729566	–	Dissanayake et al. (2020)
*Amphisphaeria sambuci*	CBS 131707^T^	NR154124	NG066215	–	MH704612	Jaklitsch et al. (2016); Liu et al. (2019)
*Amphisphaeria sambuci*	WU 33557^T^	KT949905	–	–	–	Jaklitsch et al. (2016)
*Amphisphaeria sambuci*	WU 33558	KT949906	–	–	–	Jaklitsch et al. (2016)
*Amphisphaeria shangrilaensis*	HKAS 130272^T^	NR198754	PP584776	–	–	Dissanayake et al. (2024)
*Amphisphaeria shangrilaensis*	HKAS 130273	PP584680	PP584777	–	–	Dissanayake et al. (2024)
*Amphisphaeria sorbi*	MFLUCC 13-0721^T^	NR153531	KP744475	–	–	Senanayake et al. (2015)
*Amphisphaeria thailandica*	MFLU 18-0794^T^	NR168783	MH971235	MK033640	–	Samarakoon et al. (2019)
*Amphisphaeria umbrina*	HKUCC 994	AF009805	AF452029	–	–	Jeewon et al. (2016); Jaklitsch et al. (2016)
*Amphisphaeria vemiciae*	UESTCC 23.0122	OR253155	OR253270	OR251140	–	Samarakoon (2023)
*Amphisphaeria vemiciae*	CGMCC 3.24960^T^	OR253154	OR253269	OR251139	–	Samarakoon (2023)
*Amphisphaeria xishuangbannaensis*	HKAS 130451^T^	PP584681	PP584778	–	–	Dissanayake et al. (2024)
*Amphisphaeria xishuangbannaensis*	HKAS 130452	PP584682	PP584779	–	–	Dissanayake et al. (2024)
***Amphisphaeria zhaotongensis***	**GMB6412** ^T^	**PX780726**	**PX780733**	**PX789093**	**PX794520**	**This study**
***Amphisphaeria zhaotongensis***	**GMB6413**	**PX780727**	**PX780732**	**PX789094**	**PX794521**	**This study**
*Anthostomella leucobasis*	GMB1143^T^	PP153382	–	PP198092	–	Li et al. (2024)
*Anthostomella vestita*	GMB1152^T^	PP153388	–	–	–	Li et al. (2024)
*Bartalinia pondoensis*	CBS 125525	MH863602	MH875078	MH554904	MH554421	Vu et al. (2019)
*Bartalinia pini*	CBS 143891	MH554125	MH554330	MH555033	MH554559	Liu et al. (2019)
*Crassipseudostroma phyllostachydis*	HHUF 30678^T^	NR190921	LC760577	LC760597	LC760610	Sugita et al. (2024)
*Melanographium citri*	SNC92^T^	PP592412	PP621039	PP780226	PP740449	Zhang et al. (2024)
*Melanographium palmicola*	SNC154^T^	PP592414	PP621041	PP780228	PP740451	Zhang et al. (2024)
*Melanographium phoenicis*	MFLUCC 18-1481^T^	MN482677	MN482678	–	MN481518	Hyde et al. (2020)
*Melanographium selenioides*	SNC142^T^	PP592415	PP621042	–	PP740452	Zhang et al. (2024)
*Minuticlypeus biconcavus*	GMB6221^T^	PQ874038	PQ860484	–	PQ826978	Habib et al. (2025)
*Minuticlypeus biconcavus*	GMB6222	PQ874039	PQ860485	–	PQ826979	Habib et al. (2025)
*Minuticlypeus discosporus*	HHUF 30672^T^	LC760558	LC760578	LC760598	LC760611	Sugita et al. (2024)
*Minuticlypeus discosporus*	HHUF 30673	NR190922	NG243034	–	LC760612	Sugita et al. (2024)
*Minuticlypeus rhaphidophylli*	GMB1150	PP153386	PQ860487	–	–	Habib et al. (2025)
*Minuticlypeus xiaohensis*	GMB4503	PQ066510	PQ066518	–	PQ083532	Habib et al. (2025)
*Minuticlypeus yunnanensis*	GMB5631^T^	PQ884705	PQ885417	–	–	Liu et al. (2025)
*Nigropunctata bambusicola*	MFLU 19-2134^T^	MW240662	MW240592	MW658644	MW759547	Samarakoon et al. (2022)
*Nigropunctata bambusicola*	MFLU 19-2145^T^	NR175684	NG081505	MW658646	MW759548	Samarakoon et al. (2022)
*Nigropunctata chinensis*	GMB6223^T^	PQ874034	–	PQ826932	PQ826974	Habib et al. (2025)
*Nigropunctata chinensis*	GMB6224	PQ874035	PQ860481	PQ826933	PQ826975	Habib et al. (2025)
*Nigropunctata chiangraiensis*	MFLUCC 23-0238^T^	OR909712	–	OR757300	PQ505632	Tun et al. (2024)
*Nigropunctata complanata*	HHUF 30675^T^	NR190923	NG243035	LC760600	LC760614	Sugita et al. (2024)
*Nigropunctata complanata*	HHUF 30677	LC760563	LC760583	LC760602	LC760616	Sugita et al. (2024)
*Nigropunctata hydei*	MC22-020^T^	OR507150	OR507163	OR504422	–	Samarakoon et al. (2023)
*Nigropunctata khalidii*	GMB1156^T^	PP153389	–	–	–	Li et al. (2024)
*Nigropunctata liuzhouensis*	GMB6225^T^	PQ874036	PQ860482	–	PQ826976	Habib et al. (2025)
*Nigropunctata liuzhouensis*	GMB6226	PQ874037	PQ860483	–	PQ826977	Habib et al. (2025)
*Nigropunctata nigrocircularis*	MFLU 19-2130^T^	NR175683	NG081504	–	MW759546	Samarakoon et al. (2022)
*Nigropunctata saccata*	MFLU 19-2144^T^	MW240663	MW240593	MW658645	–	Samarakoon et al. (2023)
*Nigropunctata thailandica*	MFLU 19-2118^T^	NR175682	NG081503	MW658643	MW759544	Samarakoon et al. (2022)
*Pallidoperidium chinense*	GMB6227^T^	PQ874032	PQ860478	–	PQ826972	Habib et al. (2025)
*Pallidoperidium chinense*	GMB6228	PQ874033	PQ860479	–	PQ826973	Habib et al. (2025)
*Pallidoperidium exasperatum*	HHUF 30174^T^	NR190924	NG243036	LC760603	LC760617	Sugita et al. (2024)
*Pallidoperidium exasperatum*	HHUF 30667	LC760567	LC760587	–	LC760620	Sugita et al. (2024)
*Pallidoperidium paraexasperatum*	HHUF 30668^T^	NR190925	NG243037	–	LC760621	Sugita et al. (2024)
*Pallidoperidium smilacis*	GMB1151	PP153387	PQ860486	–	–	Habib et al. (2025)
***Pallidoperidium yunnanense***	**GMB6414** ^T^	**PX782162**	**PX782156**	**PX789095**	**PX789091**	**This study**
***Pallidoperidium yunnanense***	**GMB6415**	**PX782156**	**PX782155**	**PX789096**	**PX789092**	**This study**
*Pseudoanthostomella pini-nigrae*	MFLUCC 16-0478	KX533453	KX533454	–	–	Samarakoon et al. (2022)
*Pseudoanthostomella senecionicola*	MFLUCC 15-0013^T^	MW240674	MW240604	MW658653	MW759554	Samarakoon et al. (2022)

Sequences were aligned using the MAFFT v.710 online program (Katoh et al. 2019) with default settings. The alignment was adjusted manually using BioEdit v.7.0.5.3 (Hall 1999) where necessary. The phylogeny was inferred using maximum likelihood (ML) analysis as implemented in RAxML v.8.22 using the GTRGAMMA substitution model with 1,000 bootstrap replicates (Stamatakis 2014). A second phylogenetic analysis was performed following Bayesian methodology in MrBayes v. 3.2 (Ronquist et al. 2012) online. A Markov Chain Monte Carlo (MCMC) sampling in MrBayes v.3.2.2 (Ronquist et al. 2012) was used to determine the posterior probabilities (PP). Six simultaneous Markov chains were run for 1,000,000 generations, and trees were sampled every 1,000^th^ generation. The phylogenetic tree was visualized in FIGTREE v.4.4 (Rambaut 2018). All analyses were run on the CIPRES Science Gateway v 3.3 webportal (Miller et al. 2010).

## Results

### Phylogenetic Analysis of *Amphisphaeria*

The combined sequence matrix of *Amphisphaeria* comprised 3061 (ITS: 1–501, LSU: 501–1386, *rpb2*: 1386–2206 and *tef1*: 2206–3061) characters, after exclusion of ambiguously aligned regions and long gaps. The concatenated alignment of ITS, LSU, *tef1-α* and *rpb2* comprised 3061 characters, of which 979 were parsimony-informative. *Bartalinia pondoensis* Marinc., Gryzenh. & M.J. Wingf (CBS 125525) and *B. pini* F. Liu, L. Cai & Crous (CBS 143891) were chosen as the outgroup taxa (Marincowitz et al. 2010; Liu et al. 2019). A BLASTn query of the newly generated ITS sequence (GMB6412) showed 90.4% nucleotide similarity with *Amphisphaeria ailaoshanensis* (PP584673).

**Figure 1.**
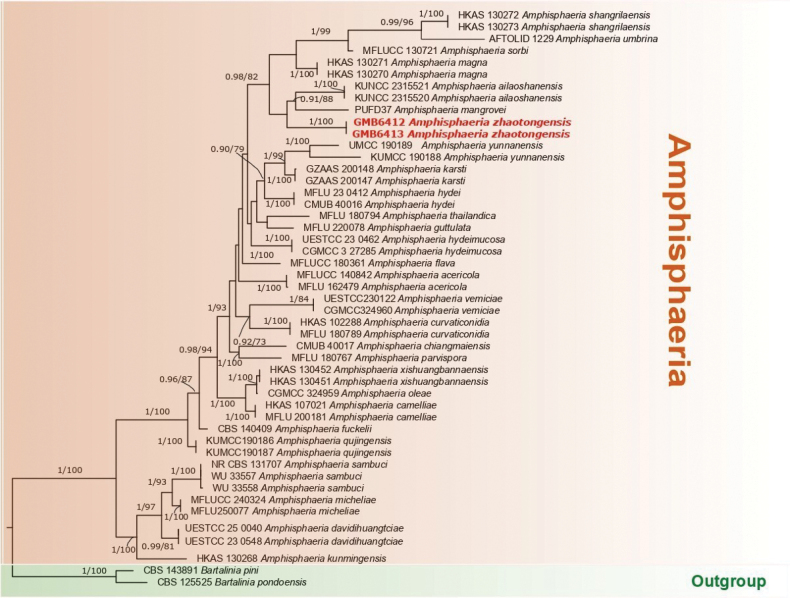
Phylogram of the best ML tree (-lnL = 18843.27) revealed by RAxML from an analysis of the combined ITS, LSU, *rpb2* and *tef-1* sequence matrix of *Amphisphaeria*. Maximum Likelihood (ML) bootstrap support values higher than 70% and Bayesian posterior probabilities (BPP) higher than 0.90 are displayed above or below the respective branches at first and second position, respectively. The newly described species is formatted in red.

The sequences of our collection *Amphisphaeria zhaotongensis* formed a clade, sister to *A. ailaoshanensis* and *A. mangrovei*. Although this relationship received low statistical support, it consistently appeared in both ML and Bayesian analyses. (Fig. 1).

### Phylogenetic Analysis of *Pallidoperidium*

**Figure 2.**
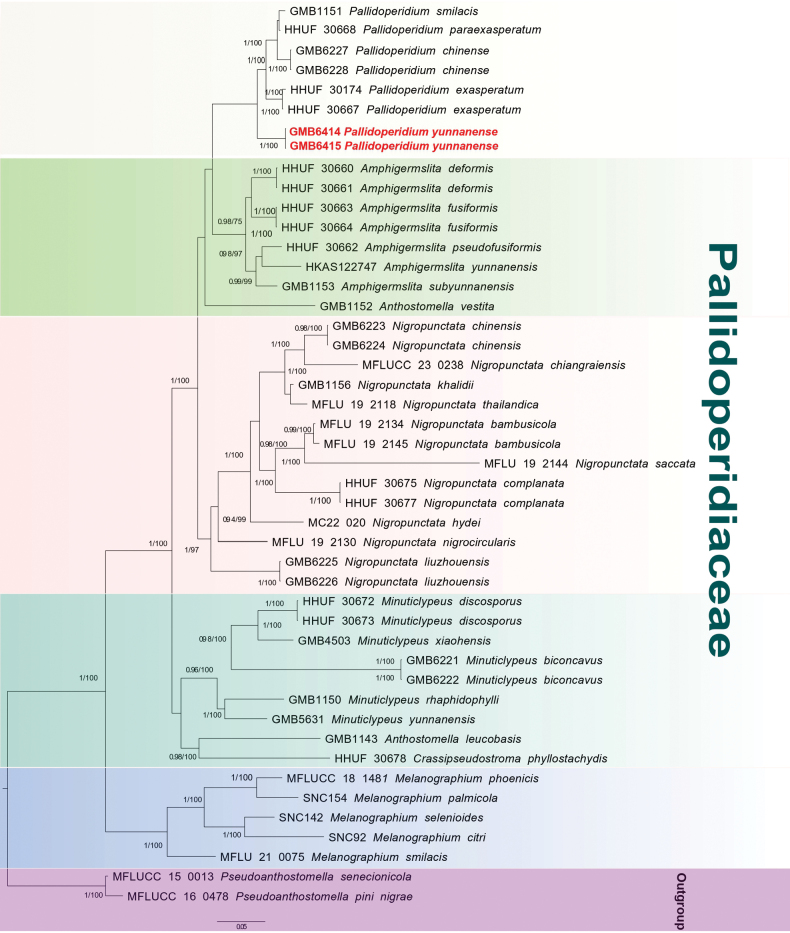
Phylogram of the best ML tree (-lnL = –20567.83) revealed by RAxML from an analysis of the combined ITS, LSU, *rpb2* and *tef-1* sequence matrix of Pallidoperidiaceae. Maximum Likelihood (ML) bootstrap support values higher than 70% and Bayesian posterior probabilities (BPP) higher than 0.90 are displayed above or below the respective branches at first and second position, respectively. The newly described species is formatted in red.

The ITS sequences extracted from our collection showed 90.4% similarity with *Pallidoperidium smilacis* (PP153387) when subjected to BLASTn query in GenBank. The combined sequence matrix of Pallidoperidiaceae (Fig. 2) comprised 3106 (ITS: 1-514 bp/198 PI, LSU: 514-1370 bp/138 PI, *rpb2*: 1370-2239 bp/240 PI and *tef1*: 2239-3003 bp/217 PI), after exclusion of ambiguously aligned regions and long gaps. *Pseudoanthostomella senecionicola* Daranag., Camporesi & K.D. Hyde (MFLUCC 150013) and *P. pini-nigrae* Daranag., Camporesi & K.D. Hyde (MFLUCC 160478) were chosen as the outgroup taxa (Daranagama et al. (2016).

In the phylogram (Fig. 2), the sequences of our *Pallidoperidium* collections (GMB6414 and GMB6415) formed a well-supported, distinct clade in a basal position within *Pallidoperidium*, supporting the recognition of a new species, described below as *Pallidoperidium yunnanense*.

### Taxonomy

#### Amphisphaeria zhaotongensis

Taxon classificationFungiAmphisphaerialesAmphisphaeriaceae

XY Luo & Q. R. Li sp. nov.

5541E9C7-CC68-5DEE-8254-D6A322C1790F

861335

Fig. 3

##### Etymology.

The specific epithet “*zhaotongensis*” refers to Zhaotong city, where the holotype specimen was collected.

##### Type.

China • Yunnan Province, Zhaotong city, Yongshan county, Wumengshan National Nature Reserve, 28°19'29.81"N, 104°00'05.54"E, altitude: 1367 m, on dead branch of an unidentified plant, June 2024, collected by Xingyu Luo, WMS162 (GMB6412, holotype; KUN-HKAS 151613, isotype; GMBC6412 ex-holotype culture).

**Figure 3.**
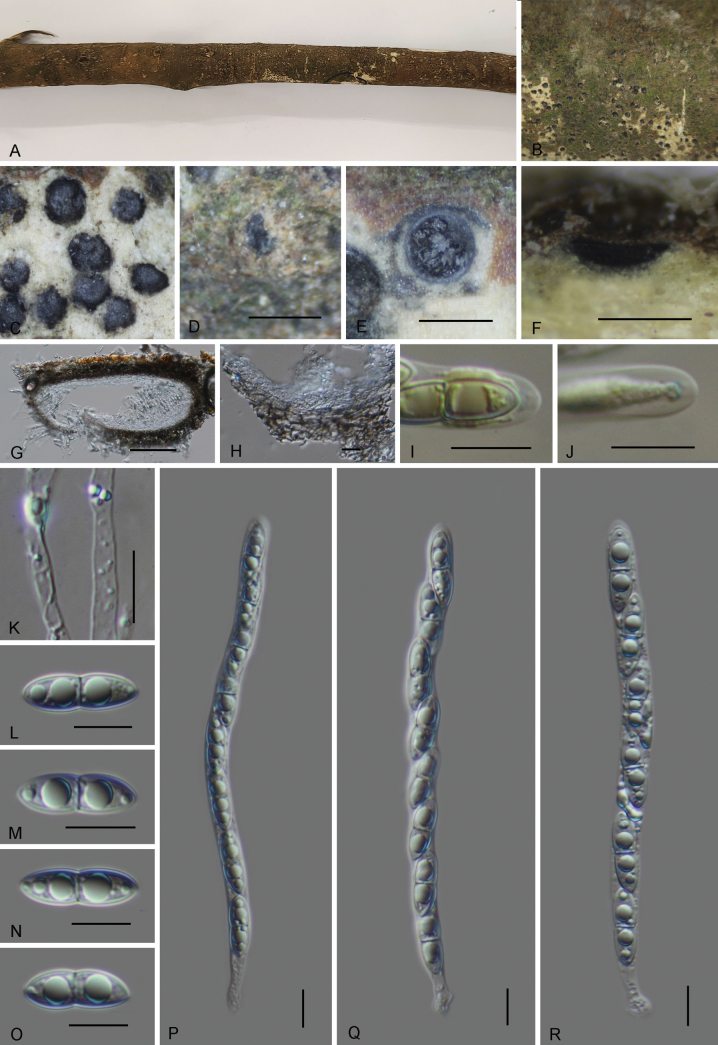
*Amphisphaeria zhaotongensis* (GMB6412, holotype). **A**. Dead corticated host branch with immersed ascomata; **B–D**. Surface view of ascomata; **E**. Cross section of ascoma; **F, G**. Vertical section of ascomata; **H**. Peridium; **I, J**. Amyloid ascal apical apparatus (stained in Melzer’s reagent); **K**. Paraphyses; **L–O**. Ascospores; **P–R**. Asci. Scale bars: 0.5 mm (**D–F**); 100 μm (**G**); 10 μm (**H–R**)

***Paratype***. China • Yunnan Province, Zhaotong city, Yongshan county, Wumengshan National Nature Reserve, 28°19'29.70"N, 104°00'11.04"E, altitude: 1322 m, on dead branch of an unidentified plant, June 2024, collected by Xingyu Luo, WMS613 (GMB6413, paratype; GMBC6413, ex-paratype culture).

##### Description.

Saprobic on the surface of a dead unknown plant. **Teleomorph**: Ascomata 350–610 µm high × 138–315 µm diam. (*x̄* = 511 × 195 µm, n = 5), perithecial, immersed in sometimes slightly uneven bark, only visible as minute dark dots, scattered or aggregated in small groups, depressed globose, dark. Peridium 32–40 µm thick, composed of several layers of pseudoparenchymatous cells, cells small to medium-sized, thin- to slightly thick-walled, brown externally and hyaline internally. Paraphyses 2.2–4.7 µm wide, hyaline, simple, multiguttulate, septate. Asci 118.5–166 × 6.9–9.3 µm (*x̄* = 138 × 7.8 µm, n = 30), 8-spored, unitunicate, cylindrical, with a short stipe, with apical apparatus 1.1–1.7 × 0.3–0.5 µm (*x̄* = 1.4 × 0.4 µm, n = 10), amyloid, bluing in Melzer’s reagent. Ascospores 17.7–21 × 4.6–6.6 µm (*x̄* = 19 × 5.9 µm, n = 30), L/W = 3.2, uniseriate, oblong or narrowly fusiform, hyaline, 1-septate at the center, slightly constricted at the septum, smooth, straight to slightly curved, thick-walled, without a mucilaginous sheath. **Anamorph**: Not observed.

##### Notes.

Phylogenetically, *Amphisphaeria zhaotongensis* is closely related to *A. ailaoshanensis* L.S. Dissan., K.D. Hyde & J.C. Kang and *A. mangrovei* Devadatha & V.V. Sarma. In BLASTn search, the ITS sequence of *A. zhaotongensis* showed highest similarity to *A. ailaoshanensis* (KUNCC 2315520, KUNCC 2315521) with 95.59%, followed by *A. mangrovei* (PUFD37) with 90.84%. The LSU sequences of *A. zhaotongensis* showed 97.51% similarity to *A. ailaoshanensis* (KUNCC 2315520, KUNCC 2315521) and 98.39% to *A. mangrovei* (PUFD37). Morphologically, *A. zhaotongensis* resembles *A. ailaoshanensis* in ascospore size but differs by having significantly larger ascomata (350–610 × 138–315 µm vs. 100–140 × 250–350 µm), longer asci (*x̄* = 138 × 7.8 µm vs. 87 × 8 µm), an amyloid apical apparatus (vs. inamyloid), and ascospores with narrow ends (vs. rounded) (Dissanayake et al. 2024).

*Amphisphaeria mangrovei* can be easily distinguished from *A. zhaotongensis* by its smaller ascomata (150–280 µm high × 140–250 µm diam vs. 350–610 µm high × 138–315 µm diam. and smaller ascospores (12–15 µm in length vs. 17.7–21 µm in length) (Phookamsak et al. 2019).

*Amphisphaeria zhaotongensis* is morphologically similar to the type species *Amphisphaeria umbrina* in having immersed, globose ascomata, cylindrical asci an amyloid apical apparatus, and 1-septate ascospores. However, it differs from *A. umbrina* in having hyaline and slightly smaller ascospores (17.7–21 × 4.6–6.6 µm), whereas *A. umbrina* has brown and slightly larger ascospores (20–25 × 6–8 µm).

#### Pallidoperidium yunnanense

Taxon classificationFungiXylarialesPallidoperidiaceae

Q. R. Li sp. nov.

E6184B00-4533-5F20-A3E0-045C54004D1C

861336

Fig. 4

##### Etymology.

The specific epithet “yunnanense” refers to the Yunnan Province, where the holotype specimen was collected.

**Figure 4.**
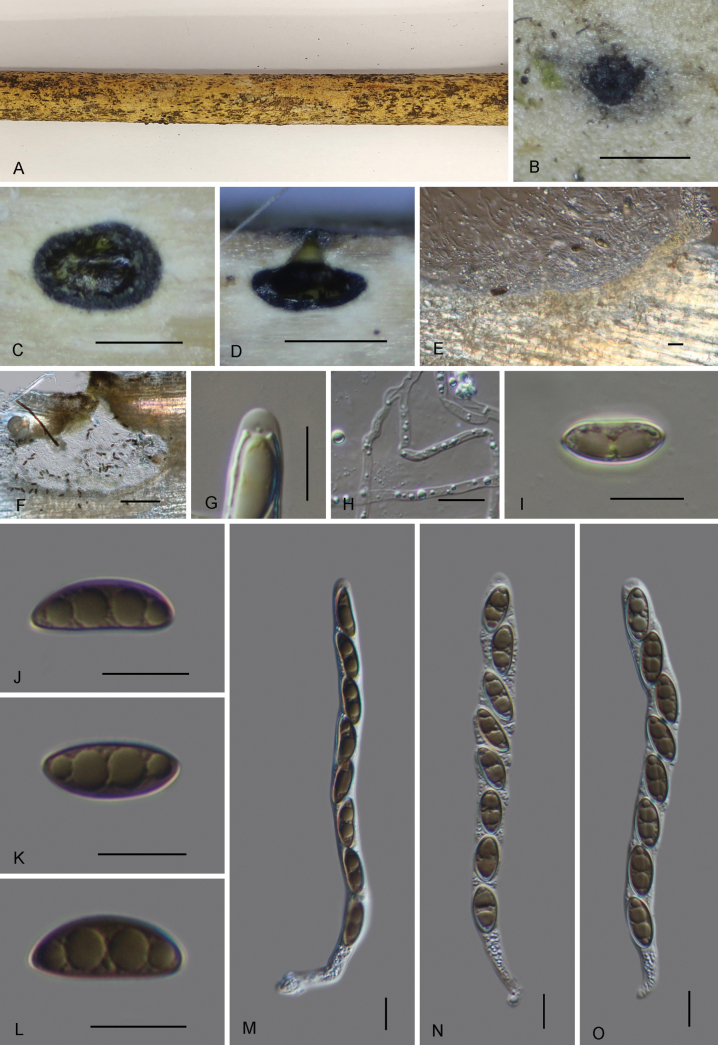
*Pallidoperidium yunnanense* (GMB6414, holotype). **A**. Dead corticated host culm with immersed ascomata; **B**. Surface view of ascomata; **C**. Cross section of ascoma; **D, F**. Vertical section of ascoma; **E**. Peridium; **G**. Inamyloid ascal apical apparatus (stained in Melzer’s reagent); **H**. Paraphyses; **I–L**. Ascospores; **M–O**. Asci. Scale bars: 0.5 mm (**B–D**); 100 μm (**F**); 10 μm (**E, G–O**)

##### Type.

China • Yunnan Province, Zhaotong city, Yongshan county, Wumengshan National Nature Reserve, 28°19'29.72"N, 104°00'11.55"E, altitude: 1228 m, on dead bamboo, June 2024, collected by Xingyu Luo, WMS149 (GMB6414, holotype; KUN-HKAS 151614, isotype; GMBC6414 ex-holotype culture).

***Paratype***. • Yunnan Province, Zhaotong city, Yongshan county, Wumengshan National Nature Reserve, 28°19'29.36"N, 104°00'10.28"E, altitude: 1300 m, on dead bamboo, June 2024, collected by Xingyu Luo, WMS614 (GMB6415, paratype; GMBC6415, ex paratype culture).

##### Description.

Saprobic on dead bamboo. **Teleomorph**: Ascomata 293–433 µm high, 446–700 µm diam., perithecial, deeply immersed in host tissue, solitary, subglobose. Ostiolar neck conical to cylindrical, 175–233 µm diam., periphysate. Ascomatal wall 15-56 µm thick, composed of 3-5 layers of elongate cells of 5–8.8 × 2–3 µm, hyaline at the inside, pale brown towards the outside. Paraphyses 1.7–3 µm wide, numerous, septate, unbranched, cylindrical, hyaline. Asci 118–151 × 6.7–10.3 µm (*x̄* = 130 × 8.5 µm, n = 20), 8-spored, unitunicate, cylindrical, with a short stipe, with apical apparatus not bluing in Melzer’s reagent. Ascospores 14.4–18 × 5.5–7.3 µm (*x̄* = 16 × 6.4 µm, n = 30), l/w = 2.5, uniseriate, ellipsoid to fusiform, unicellular, brown, surrounded by a narrow mucilaginous sheath. **Anamorph**: Not observed.

##### Habit and habitat.

Scattered on dead bamboo.

##### Notes.

Morphologically, *Pallidoperidium yunnanense* resembles *P. exasperatum* R. Sugita & Kaz. Tanaka and *P. paraexasperatum* R. Sugita & Kaz. Tanaka, in having immersed perithecial ascomata, cylindrical unitunicate asci with a discoid apical apparatus, and brown, ellipsoid to fusiform ascospores. However, *P. yunnanense* differs from *P. exasperatum* by having markedly broader ascomata (446–700 µm vs. 320–390 µm wide) and ascospores with a narrow mucilaginous sheath (vs. ascospores with a thick mucilaginous sheath) (Sugita et al. 2024).

*P. yunnanense* is distinguished from the additional two species in the genus by its smaller, narrower ascospores (14.4–18 × 5.5–7.3 µm) compared to *P. chinense* (18–35 × 5–9.5 µm) and *P. smilacis* (16.3–18.3 × 7.7–8.4 µm). In addition, *P. chinense* and *P. smilacis* have smooth to roughened ascospores with a thick mucilaginous sheath, while mucilaginous sheath narrow in *P. yunnanense* (Habib et al. 2025).

Phylogenetically, *Pallidoperidium yunnanense* forms an independent clade in a basal position within the genus. Although phylogenetically distinct, its morphology is consistent with the generic concept of *Pallidoperidium*, including subglobose, deeply immersed perithecial ascomata, discoid apical apparatus, and ellipsoid to fusiform brown ascospores (Table 2).

**Table 2.** Distinguishing characters of *Pallidoperidium* species.

Character	*Pallidoperidium yunnanense*	*P. exasperatum*	*P. paraexasperatum*	*P. chinense*	*P. smilacis*
Ascomata size	293–433 µm height × 446–700 µm diam.	300–440 µm height × 320–390 µm diam.	290–350 µm height × 320–340 µm diam.	300–500 µm height × 350–600 µm diam.	412–443 µm height × 292–443 µm diam.
Ascospore size	14.4–18 × 5.5–7.3	15.5–22.5 × 6–7.5	15–19 × 5.5–7.5	18–35 × 5–9.5	16.3–18.3 × 7.7–8.4
Ascospore ornamentation	Rough, narrow mucilaginous sheath	Rough, with thick mucilaginous sheath	Rough, with mucilaginous sheath	Smooth to rough, thick mucilaginous sheath	Smooth, with mucilaginous sheath
Apical apparatus	Inamyloid	Amyloid, thin discoid	Amyloid, thin discoid	Inamyloid	Inamyloid

## Discussion

Southwestern China is considered a hotspot for diverse fungal taxa, especially for wood-inhabiting fungi which contribute considerably to nutrient cycling and ecosystem balance (Hyde et al. 2020; Samarakoon et al. 2022). Here, we describe two novel taxa from this unique habitat, namely, *Amphisphaeria zhaotongensis* and *Pallidoperidium yunnanense*, characterized and described by using both a morphological and multi-gene phylogenetic approach. These discoveries underscore the still hidden fungal diversity of this region and provide insights into phylogenetic relationships within Xylariomycetidae.

Species of *Amphisphaeria* are usually saprobes on woody branches of different hosts, distributed in diverse ecological zones, such as grasses in terrestrial, mangrove, and freshwater habitats (Senanayake et al. 2019; Samarakoon et al. 2020; Samarakoon et al. 2022; Dissanayake et al. 2020). However, *A. orixae* was isolated as an endophyte from the medicinal plant *O. japonica* (Wang et al. 2023).

Species of *Amphisphaeria* are morphologically distinguished by immersed to erumpent ascomata, unitunicate asci with an apical apparatus that is amyloid or inamyloid, and often hyaline to brown ascospores (Samarakoon et al. 2020; Wang et al. 2023; Dissanayake et al. 2024); however, these characteristics frequently overlap across species. Due to significant morphological overlap among species and by its historical ambiguity with the genus *Lepteutypa*, which was previously distinguished primarily on stromatal form and ascospore septation but which cannot be phylogenetically separated from *Amphisphaeria*, the genus *Amphisphaeria* has undergone numerous taxonomic rearrangements (Samarakoon et al. 2020; Senanayake et al. 2015).

Our phylogenetic analysis confirms the placement of *A. zhaotongensis* within *Amphisphaeria*, forming a distinct lineage among recently described Chinese species. The discovery of this species in subtropical forests contributes to our understanding of the genus’s diversity in the region and highlights the value of multilocus approaches for reliable species identification. To date, Species Fungorum recognizes approximately 169 species of the genus (accessed September 18, 2025). Prior to this study, *Amphisphaeria* species reported from China include *A. ailaoshanensis*, *A. xishuangbannaensis*, *A. yunnanensis*, *A. karsti*, *A. shangrilaensis*, *A. qujingensis*, *A. kunmingensis*, *A. davidihuangtciae*, *A. hydeimucosa* (Dissanayake et al. 2020, 2024; Du et al. 2025; Habib et al. 2025). With the addition of *A. zhaotongensis*, the known diversity of the genus in China is further expanded.

The family Pallidoperidiaceae was recently introduced to accommodate and resolve a longstanding ambiguity for the placement of several *Anthostomella*-like taxa (Sugita et al. 2024). Prior to this study, four species were recognized worldwide: *P. exasperatum*, *P. paraexasperatum* from Thailand and *P. chinense*, *P. smilacis* from China (Sugita et al. 2024; Habib et al. 2025).

All known species of *Pallidoperidium*, including the newly described *P. yunnanense*, have been collected exclusively from bamboo substrates indicating a strong host preference of this genus (Sugita et al. (2024); Habib et al. (2025). Such host specificity may indicate an ecological adaptation to the lignocellulosic composition or microenvironmental conditions of bamboo culms. Moreover, additional sampling from additional bamboo hosts and a wider geographical area is required to confirm whether *Pallidoperidium* is an exclusively bamboo-associated lineage or simply a group with strong ecological affinity for bamboo habitats.

The addition of *Pallidoperidium yunnanense* and *Amphisphaeria zhaotongensis* highlights the taxonomic and ecological diversity of Yunnan’s Xylariomycetidae. Resolving taxonomic boundaries within Amphisphaeriaceae and Pallidoperidiaceae will require further comprehensive studies that combine morphology with multigene phylogenetic frameworks, as many species lack molecular data and several allied species are still poorly defined.

## Supplementary Material

XML Treatment for Amphisphaeria zhaotongensis

XML Treatment for Pallidoperidium yunnanense

## Additional information

### Use of AI

No use of AI was reported.

### Author ORCIDs

Ming-Hui He https://orcid.org/0009-0005-2395-7395

Xing-Yu Luo https://orcid.org/0009-0004-8077-4272

Kamran Habib https://orcid.org/0000-0003-2572-0306

Ping-Zhu Lu https://orcid.org/0009-0005-2612-4110

Yi-Lan Jin https://orcid.org/0000-0001-8803-1036

Urooj Ashraf https://orcid.org/0009-0008-9624-0813

Qi-Rui Li https://orcid.org/0000-0001-8735-2890

### Data availability

All of the data that support the findings of this study are available in the main text.
